# Breeding for disease resilience: opportunities to manage polymicrobial challenge and improve commercial performance in the pig industry

**DOI:** 10.1186/s43170-022-00073-y

**Published:** 2022-01-15

**Authors:** Xuechun Bai, Graham S. Plastow

**Affiliations:** Livestock Gentec, Department of Agricultural, Food and Nutritional Science, University of Alberta, Edmonton, AB Canada

**Keywords:** Disease resistance, Disease tolerance, Disease resilience, Infectious disease

## Abstract

Disease resilience, defined as an animal’s ability to maintain productive performance in the face of infection, provides opportunities to manage the polymicrobial challenge common in pig production. Disease resilience can deliver a number of benefits, including more sustainable production as well as improved animal health and the potential for reduced antimicrobial use. However, little progress has been made to date in the application of disease resilience in breeding programs due to a number of factors, including (1) confusion around definitions of disease resilience and its component traits disease resistance and tolerance, and (2) the difficulty in characterizing such a complex trait consisting of multiple biological functions and dynamic elements of rates of response and recovery from infection. Accordingly, this review refines the definitions of disease resistance, tolerance, and resilience based on previous studies to help improve the understanding and application of these breeding goals and traits under different scenarios. We also describe and summarize results from a “natural disease challenge model” designed to provide inputs for selection of disease resilience. The next steps for managing polymicrobial challenges faced by the pig industry will include the development of large-scale multi-omics data, new phenotyping technologies, and mathematical and statistical methods adapted to these data. Genome editing to produce pigs resistant to major diseases may complement selection for disease resilience along with continued efforts in the more traditional areas of biosecurity, vaccination and treatment. Altogether genomic approaches provide exciting opportunities for the pig industry to overcome the challenges provided by hard-to-manage diseases as well as new environmental challenges associated with climate change.

## Background

As pork is one of the most commonly consumed meats in the world, economies of scale and high demand for meat, driven by population growth, have led to intensification and consolidation of pig production. Globalization also contributes to an increasing movement of pigs, feed, and pork products on local, national, and international scales. Within this framework, endemic and emerging pathogens can be spread rapidly in commercial pig farms by common farm activities and can result in severe and even catastrophic consequences. The current challenge of disease in the pig industry is caused by a multitude of infectious pathogens that exist around the world. For example, porcine reproductive and respiratory syndrome virus (PRRSV), porcine circovirus type 2 (PCV2), influenza A virus, *Salmonella* spp., and *Escherichia coli* (*E. coli*) were recognized as the most important pathogens which have been reported from nearly every country producing pigs (VanderWaal and Deen 2018). In addition, African swine fever (ASF) virus and porcine epidemic diarrhea (PED) virus have become increasingly important pathogens, although they have not yet spread globally. Severe outbreaks of ASF have been reported in multiple countries across Africa, Europe, and more recently, China and other Asian countries (Cwynar et al. 2019; Dixon et al. 2019; Sánchez-Vizcaíno et al. 2015; Zhou et al. 2018). PED has emerged and re-emerged in European, East Asian, and American countries (Hanke et al. 2017; Lara-Romero et al. 2018; Lv et al. 2016; Ojkic et al. 2015; Sun et al. 2001; Wang et al. 2014). It was estimated that China alone had experienced direct economic losses of US$ 141 billion from August 2018 to September 2019 because of ASF (Berthe 2020). PED was reported to have caused the loss of 7 million pigs within 12 months of its introduction to the USA in 2013 (Niederwerder and Hesse 2018).

Infectious diseases have steadily increased morbidity and mortality in multiplication and production herds of the pig industry, which causes a significant loss of productivity. Moreover, infectious diseases also threaten food safety, animal welfare, and cause international trade restrictions to the industry (Davies et al. 2009; Tomley and Shirley 2009). The constant threats of infection have resulted in significant economic losses to the pig industry, which in some instances (e.g. Influenza, *Streptococcus suis*, *Salmonella* spp., *E. coli*) also impacts human health (Alarcon et al. 2013; Evangelopoulou et al. 2015; Gillespie et al. 2009; Holtkamp et al. 2013; Honish et al. 2017; Mason-D’Croz et al. 2020; Nieuwenhuis et al. 2012; Tseng et al. 2014). The uncontrolled use of antimicrobials to treat diseases in pigs will contribute to the global challenge of antimicrobial resistance in addition to zoonoses (O’Neill 2016). Moreover, co-infections associated with multiple infectious agents are more frequent in pig farms and may contribute to severe and long-term problems in pigs reared under confined production conditions. For example, the porcine respiratory disease complex caused by multiple viruses (e.g. PRRSV, PCV2, swine influenza virus, coronavirus) and bacteria (e.g. *Mycoplasma hyopneumoniae*, *Haemophilus parasuis*, *Streptococcus suis*, *Bordetella bronchiseptica*, *Actinobacillus suis*, *Actinobacillus pleuropneumoniae*) is one of the most common conditions founds in intensive production (Kavanová et al. 2017; Opriessnig et al. 2011; Ouyang et al. 2019; Saade et al. 2020).

Therefore, the prevention and control of infection in herds across the production pyramid are essential for animal and human health, animal welfare, and maintaining the productivity and sustainability of the pig industry. Conventional methods, including strict biosecurity, vaccines, and antimicrobials, have been used to prevent and control infection but are not always effective. The diverse ways for pathogen transmission and the gaps in knowledge of epidemiology for diseases, especially those caused by the emergence of new pathogens into farms and countries currently not affected (e.g. PED virus to the United States in 2013; ASF virus emerging in China in 2018), are challenges for successful biosecurity (Stevenson et al. 2013; Zhou et al. 2018). As indicated globalization also makes it more challenging for prevention and the exclusion of specific pathogens from territories and regions. The high evolution and recombination rate for some viruses (e.g. PRRSV) and their immunosuppressive properties make it hard to develop effective vaccines (Nan et al. 2017; Thanawongnuwech and Suradhat 2010). For example, highly pathogenic forms of PRRSV have occurred in China since 2006 and multiple new variants consistently contribute to a large number of abortions and death in pig farms (Dong et al. 2018; Song et al. 2020; Tong et al. 2007; Yu et al. 2018; Zhao et al. 2015). In the last quarter of 2020, a highly pathogenic PRRSV 1-4-4 variant strain emerged and is now severely impacting pig production through the Midwest region of the US (Trevisan et al. 2021). In addition, co-infection with other pathogens also impairs vaccine efficacy, although it protects the host from infection in the absence of other pathogens. For example, the presence of PRRSV can cause a significant reduction in the efficacy of the swine influenza virus vaccine (Kitikoon et al. 2009). The occurrence and concern of antimicrobial resistance require the pig industry to limit the use of antimicrobials by employing principles of antimicrobial stewardship and utilizing alternatives where possible (Lammie and Hughes 2015). Overall, the impact of infectious disease and the concern with the breakdown of conventional disease control methods highlight the potential vulnerability of the swine industry and the urgency of preventing and managing infectious diseases. The global nature of the industry together with the emergence and re-emergence of difficult-to-control diseases mean that whilst strict biosecurity and vaccination will continue to play a key role in disease prevention and management, they need to be supported by additional approaches that improve the effectiveness of infectious disease control in the swine industry.

One component that is relatively overlooked in terms of the management of pig health is the selection of animals that are less susceptible to infectious disease. Genetic improvement for host response to infection is proposed as a complementary strategy to help the pig industry cope with this problem of infectious disease in addition to the more traditional approaches of biosecurity, vaccination and treatment. One of the challenges for this approach is the collection of data to be able to estimate the breeding value of animals. There is a significant difference between selection and production environments as breeding companies need to ensure elite breeding stock are selected under a biosecure and high-health environment to allow dissemination of genetic improvement in the absence of diseases. Meanwhile, they also need the offspring of the selected animals to express their genetic potential and perform in a disease-challenged production environment (Neeteson-van Nieuwenhoven et al. 2013). Although selection can be made for commercial performance using variants of progeny testing where phenotypes measuring disease response in commercial herds are collected, these approaches are expensive and difficult to organize as they have required single-sire mating and accurate recording at the commercial level (see Newman et al. 2010). The results will depend on the level of disease challenge in the different farms as well as the different pathogens present. As there is genetic variation in resistance (an animal’s ability to maintain health or restrict the proliferation of pathogens and reduce within-host pathogen burden) to almost all pig pathogens, there is the potential to dissect and select for genetic resistance to infectious diseases (Davies et al. 2009; Plastow 2016). Indeed, PIC used simultaneous collection of purebred and crossbred records from nucleus sires to improve disease robustness as measured by grow-finish mortality (Newman et al. 2010). However, the problems associated with using mortality are set out by Knap and Doeschl-Wilson (2020). More recently, the breeding company, Topigs Norsvin has implemented selection for increased natural resistance to PRRSV in the breeding program (Topigs 2018). PIC is currently exploring the use of gene edited pigs to deliver resistance to PRRSV (Burkhard et al. 2017; PIC 2021). However, as there are many different pathogens impacting pig health and performance it may be an endless task to take this approach, although it may play a role for the major diseases such as PRRS and *E. coli* associated scours that were strongly justified as targets for genomic studies (Davies et al. 2009), and potentially for diseases such as ASF in the future. Alternatively, disease resilience, defined as an animal’s ability to maintain high production levels despite disease and potentially applicable to multiple pathogens, has been identified as a desirable breeding goal and trait for pig breeding programs (Albers et al. 1987; Harlizius et al. 2020; Mulder and Rashidi 2017). However, no breeding company has carried out selection for increased disease resilience to multiple infectious pathogens in the breeding scheme to date, which may be due to two significant obstacles. Firstly, the terminology around disease resilience and its component traits of disease resistance and tolerance may be confusing and must be clarified and unified. Secondly, disease resilience is a complex trait that consists of multiple biological functions (e.g. production, reproduction, and immune responses) and dynamic elements of rates of response and recovery from infection, which can be hard to characterize thoroughly (Friggens et al. 2017). Therefore, new, easy and inexpensive traits for practical breeding of disease resilience need to be explored and developed.

In line with the above, this paper has three objectives. Firstly, to refine the definitions of disease resilience and its component traits of disease resistance and tolerance based on previous studies to improve the understanding and application of these breeding goal traits under different scenarios. Secondly, to describe and summarize our own efforts and those of our colleagues to explore traits of disease resilience for practical pig breeding from a “natural disease challenge model”. Lastly, to discuss the way forward for application of these approaches for improving the productivity and sustainability of the pig industry.

## Main text

### Disease resistance

Disease resistance has been invariably discussed as a strategy for infectious disease control, but multiple studies have different interpretations of the mechanisms and approaches available. Therefore, it is helpful to clarify and unify the mechanism of disease resistance in terms of its role in epidemiology and animal breeding. Here, “narrow sense” and “broad sense” definitions of disease resistance are used to distinguish and define resistance from a livestock viewpoint.

### The narrow sense definition of resistance

Disease resistance can be achieved by avoiding infection (entrance and development of infectious agents in the body of an animal) in the first place. This is regarded here as a “narrow sense” definition of disease resistance because it describes a particular situation where an animal has the ability to maintain a completely healthy status, so called complete resistance, when challenged by infectious agents. The most apparent cause of such resistance is the absence of receptors on the target tissue or cell required for the pathogen to attach and infect or produce toxins that impact the health and performance of an animal. This failure of the pathogen to attach or adhere to the receptor stops the very first step of host–pathogen interaction and prevents infection or transfer of toxic compounds (Plastow 2016). Two examples of such genetic resistance in pigs are related to scours caused by *E. coli* F18 and F4 (or K88) (Augustino et al. 2020; Bao et al. 2012; Fu et al. 2012; Meijerink et al. 2000; Meijerink et al. 1997; Ren et al. 2012; Zhang et al. 2008). Such resistance is the most cost-effective mechanism of preventing infection from the host perspective as there is no need to increase energy expenditure on the immune system for defending against infectious agents when the resistant animal can avoid infection and maintain a healthy status. However, this assumes that the absence of the receptor does not impact other important functions involved in pig performance. For example, attention has been paid to the production impact of the AA genotype in the alpha-(1,2) fucosyltransferase (*FUT1*) gene, which results in complete resistance to *E. coli* F18. Results suggested that the AA genotype is also a beneficial genotype for meat quality, growth, development and reproductive performance (Bao et al. 2011). In addition, *FUT1* genotype may potentially be associated with the gut homeostasis and plasma metabolic profile of piglets pre and post-weaning. Further investigation is warranted as these aspects could affect the intestinal ecosystem and related gut metabolism, inflammation and nutrient absorption capability (Poulsen et al. 2018). The neutral or positive effects on production traits as seen with *FUT1* may not necessarily always be the case. For example, resistance to *E. coli* F4 was often reported to be associated with reduced growth performance (Edfors-Lilja et al. 1986). However, a recent study suggests this may not be a concern as there was no significant difference observed between resistant and susceptible pigs for 3 weeks after challenge (Roubos-van den Hil et al. 2017).

Another aspect that is important here is the absence of variation in the receptor so that selective breeding for this type of resistance is constrained by the standing genetic variation. In this case, gene-editing technologies offer new opportunities for creating new variants of genes involved in host–pathogen interactions, such as the modification of genes that encode receptors involved in the initial steps of disease. CRISPR/Cas9 originally identified as an adaptive immune system in bacteria to defend against the invasion of foreign genetic elements through DNA or RNA interference, has been adapted as a high efficiency and low cost tool for gene-editing (Doudna and Charpentier 2014). Perhaps the most successful example of gene-editing in pigs to date is the modification of the *cluster of differentiation 163* (*CD163*) gene to generate pigs completely resistant to PRRSV. Briefly, CD163 is a scavenger receptor expressed on peripheral blood monocytes and macrophages. A major function of CD163 is the “hemoglobin scavenger receptor”, including the recognition and endocytosis of hemoglobin-haptoglobin complexes from the circulation and re-use of haem to prevent oxidative toxicity of free hemoglobin and is an important anti-inflammatory function of CD163 (Kristiansen et al. 2001). In addition, CD163 is also an important component of PRRSV infection of porcine alveolar macrophages (Calvert et al. 2007). The knockout or modification of specific domains of CD163 result in complete resistance to PRRSV. Those animals with CD163-null phenotype macrophages generated by the knockout of the *CD163* gene were completely resistant to several isolates of both type 1 and type 2 PRRSV (Wells et al. 2016; Whitworth et al. 2016). However, the knockout of the *CD163* gene may have a negative impact on animals due to the important role of CD163 in scavenging haemoglobin. Susequently, a precision modification was used to only delete Exon 7 of the *CD163* gene, encoding the scavenger receptor cysteine-rich domain 5 (SRCR5). The SRCR5 is an interaction site for PRRSV infection with no other known biological functions (Burkard et al. 2018; Burkard et al. 2017; Wells et al. 2016). Modified pigs lacking the CD163 SRCR5 domain were fully resistant to both type 1 and type 2 PRRSV genotypes, and no adverse effects were identified on growth rate or immune cell counts of the gene-edited pigs (Burkard et al. 2017). CRISPR/Cas9 was also used to generate pigs resistant to transmissible gastroenteritis virus (TGEV), a globally distributed disease associated with large economic losses in pork production, by removing its receptor, aminopeptidase N (Whitworth et al. 2019). In terms of the polymicrobial challenge in the pig industry, these two edits have then been combined to generate pigs resistant to both viruses, PRRSV and TGEV (Xu et al. 2020). The double-edited pigs were reported to have no differences in production or reproductive-performance traits compared to wild-type pigs, although a higher iron content was found in their muscles which led to a significant increase in meat color score (redness) (Xu et al. 2020).

Given the high efficiency and low cost of genome editing tools, particularly CRISPR/Cas9, gene-edited pigs seem to hold great promise for the future production of animals resistant to diseases over a shorter time-period. However, this may not be the case if the function of the receptor is essential for the host so that edits or knockouts would be lethal. Moreover, the pathogen may use multiple different receptors to initiate interaction with the host and subsequent steps in infection, which challenges the creation of complete disease resistance using gene-editing tools. The efficacy of genome editing may also be time-limted due to the evolution and emergence of escape variants of pathogens, similar to the risk associated with vaccines (Kimman et al. 2009; Tait-Burkard et al. 2018). For example, this may be a justified concern for PRRSV, an RNA virus with a high mutation rate (Tait-Burkard et al. 2018). Therefore, the potential side effects and the efficacy associated with genome editing for more complex situations in the pig industry will need to be closely monitored and explored. In addition, ethical and welfare concerns with genome editing should not be overlooked because the probability of obtaining a live genome edited animal is not high at the moment (Bastiaansen et al. 2018). Such low probability can be caused by either low survival of edited zygotes or the occurrence of off-target effects (nonspecific and unintended genetic modifications) and mosaicism (the presence of more than one genotype in one individual because the CRISPR/Cas9 system can continuously target and cleave genes at different stages of embryonic development) (Bastiaansen et al. 2018; Carroll 2019). Off-target and mosaic genome edited animals do not survive or cannot be used for breeding due to ethical and safety reasons (Bastiaansen et al. 2018). Moreover, uncertainty about consumer acceptance and the regulatory framework are also major hurdles in implementing gene-editing technologies. Approval of gene-edited pigs for human consumption relies on national and international legislation, which is currently still at an early stage (Proudfoot et al. 2019). Last, but not least, a careful assessment of the cost and benefits of genome editing is also essential before it can be further developed in commercial livestock (Bastiaansen et al. 2018).

### The broad sense definition of resistance

Back to the strategy of selective breeding for resistance, since the selection of complete resistance is constrained by the standing variation, the term “disease resistance” is often loosely used as an animal’s response after the infection has been established. Therefore, once the infection is established, the host defense strategy is termed disease resistance in a broad sense and defined as an animal’s ability to employ immune responses which work by detection, neutralization, and destruction of pathogens to restrict the proliferation of pathogens and reduce within-host pathogen burden (Bishop 2012; Bishop and Morris 2007; Bishop and Stear 2003; Bishop and Woolliams 2014). Candidate resistance genes are expected to encode molecules associated with immune responses, which lead to pathogen load reduction or even pathogen clearance (Glass 2012). Although such animals are not completely resistant in this case, improving such disease resistance may have the potential to reduce disease prevalence as the effect on reducing pathogen burden could benefit other susceptible population members by reducing the transmission of infection.

To date, the broad sense definition of disease resistance has been recognized in multiple studies to be a relative rather than an absolute status as is the case for the narrow sense definition of disease resistance. In order to compare the level of this type of disease resistance (broad sense definition) among animals, pathogen burden, such as fecal egg count, viremia (viral load), or bacterial load, needs to be measured for animals infected with parasites, viruses, or bacteria, respectively (Bishop 2012). This is typically expensive to implement in commercial production. In pigs, a major focus has been on PRRSV as it is the causative agent of a major endemic disease globally and the existence of genetic basis of disease resistance to PRRSV infection was first found at a breed level. For example, a Hampshire × Duroc synthetic line was found to have higher viremia at 4, 7, and 14 dpi with PRRSV than a Yorkshire × Landrace line (Petry et al. 2005). Subsequently, higher PRRSV viremia was observed in Pietrain pigs than in Yorkshire pigs (Doeschl-Wilson et al. 2009). Later on, genome-wide association studies identified a genetic variation in the resistance of pigs to PRRSV infection, with a single-nucleotide polymorphism (SNP, WUR10000125, so called “WUR”) on chromosome 4 explaining approximately 15% of the genetic variance for viral load (Boddicker et al. 2012, 2014a, b). Further studies found a truncated *GBP5* (encoding guanylate-binding protein 5) variant was associated with the AA genotype at the WUR locus, which is the unfavourable genotype and may reduce an animal’s ability to inhibit viral entry and replication as the GBP5 protein was previously shown to play a role in immune response through mediation of inflammasome assembly (Koltes et al. 2015; Schroyen et al. 2016; Shenoy et al. 2012). Later on, Dunkelberger et al. (2017) further estimated the effect of WUR in commercial pig lines and indicated that selecting for the favorable (B) allele at WUR SNP can improve resistance to PRRS in progeny without compromising overall economic value under nonchallenging conditions. Similar results were reported by Zhang et al. (2020).

Before applying selective breeding for broad sense disease resistance in practice it is important to be aware of the potential for increased host resistance to fuel the “arms race” between host and pathogen and stimulate pathogen evolution and mutation toward higher virulence and multiple ways of evasion from the host immune system (Doeschl-Wilson and Kyriazakis 2012). Furthermore, most studies of disease resistance are pathogen-specific, and the genetic basis for animals to be disease resistant under a polymicrobial challenge in the field remains mostly unknown. There is a concern that the selective breeding of pigs to be more disease resistant to a specific type of pathogen may have some serious drawbacks for their health due to the inadvertent increase of susceptibility to other pathogens. For example, the selection of resistant animals with a strong humoral-mediated immune response to extracellular pathogens might inadvertently increase their susceptibility to intracellular pathogens controlled by cell-mediated immune responses due to the inverse relationship and trade-off between antibody production and macrophage activity (Hine et al. 2014; Thompson-Crispi et al. 2012).

### Disease tolerance

Another concept that relates to reduced disease susceptibility is disease tolerance. Disease tolerance is defined as an animal’s ability to mitigate the detrimental impact of infection on the host performance and fitness caused by the toxicity of pathogens and immunopathology under a given pathogen burden but does not exert any direct negative effect on the pathogen itself (Ayres and Schneider 2012; Bishop 2012; Doeschl-Wilson et al. 2012b; Nakov et al. 2019). This latter point means that tolerance would not be a concern in terms of the “arms race” between host and pathogen and escape variants. For this reason disease tolerance has been suggested as an alternative breeding goal trait for the pig industry to cope with the infection. The current understanding of disease tolerance mechanisms is limited but seems to revolve around responses that confer tissue damage control and repair to maintain homeostasis and functional integrity of tissues and organs in the infected host (Shourian and Qureshi 2019; Soares et al. 2014, 2017). Thus, candidate genes that function as regulators of the intensity and duration of the host inflammatory response may be involved in the establishment of disease tolerance (Glass 2012). During the infection, the toxicity caused by the pathogen itself is not the only detrimental impact of infection, immunopathology may result from collateral damage caused by the immune mechanisms that provide defense against pathogens and this can be a significant concern in some cases (Glass 2012). For example, macroscopic lesions in organs have been consistently observed in pigs infected by ASF virus, and most lesions in such cases can be attributed to the release of cytokines by infected monocytes and macrophages rather than by virus-induced direct cell damage (Blome et al. 2013; Netherton et al. 2019; Sánchez-Vizcaíno et al. 2015). Furthermore, the ability to control and repair tissue damage caused by host immune responses and the pathogenicity of the infectious agent could also be an essential component of disease tolerance (Glass 2012; Medzhitov et al. 2012; Medzhitov 2009).

Thus, tolerance mechanisms are expected to be more host rather than pathogen-specific, as they do not directly impact the pathogen burden within the host. Improving disease tolerance may have a neutral effect or even stimulate the prevalence of the pathogen. This is because tolerant animals can harbour a high pathogen load and potentially act as “super-spreaders” to infect larger numbers of susceptible animals on the farm, or on a national and international scale (McCarville and Ayres 2018). However, it may be more beneficial to improve disease tolerance than resistance when individuals are exposed to multiple pathogens in commercial pig farms with a high risk of pathogen evolution and the difficulty of disease eradication due to the presence of asymptomatic carriers (Doeschl-Wilson and Kyriazakis 2012). Even so, asymptomatic genetically tolerant individuals within a mixed population containing susceptible animals could increase disease incidence and prevalence (Tait-Burkhard et al. 2018).

Reaction norms are used to determine and target disease tolerance for genetic improvement. This term originates from ecology and describes the pattern of phenotypic expression across a range of environments and in this case pathogen burdens (Råberg et al. 2007; Simms 2000). One approach to measure tolerance is to determine the change in host production performance with respect to the change in within-host pathogen burden. Random regression models have been typically used for estimating individual tolerance by repeated measures of host performance and pathogen burden at multiple time points where the host performance records (y-axis) are regressed against the measures of pathogen burden (x-axis), and the slope refers to the level of individual tolerance (Kause 2011; Kause and Odegård 2012; Lough et al. 2017, 2018). For example, genetic variation in tolerance of pigs to PRRSV infection has been investigated using random regression models with repeat measurements of viral load and average daily gain throughout the infection (Lough et al. 2017, 2018). This showed that genetic variance in tolerance of pigs to PRRSV infection may exist but its effect is relatively small (Lough et al. 2017). Interestingly, the major locus affecting resistance to PRRSV challenge, WUR on chromosome 4, was also found to be significantly associated with tolerance to PRRSV infection (Lough et al. 2018).

Using random regression models for estimating genetic parameters and breeding values for disease tolerance is particularly attractive as they can be readily and easily applied. However, random regression models assume host tolerance is constant. In other words, the impact associated with a given pathogen burden does not change over time, which is not always the case in practice. In addition, the host’s ability to contain and remove a pathogen through immune responses (the broad sense of disease resistance) exerts effects on both phenotypic expression of tolerance and pathogen burden, suggesting that the interaction between disease resistance and tolerance should not be ignored.

### Disease resilience: resistance and tolerance interactions

**Fig. 1:**
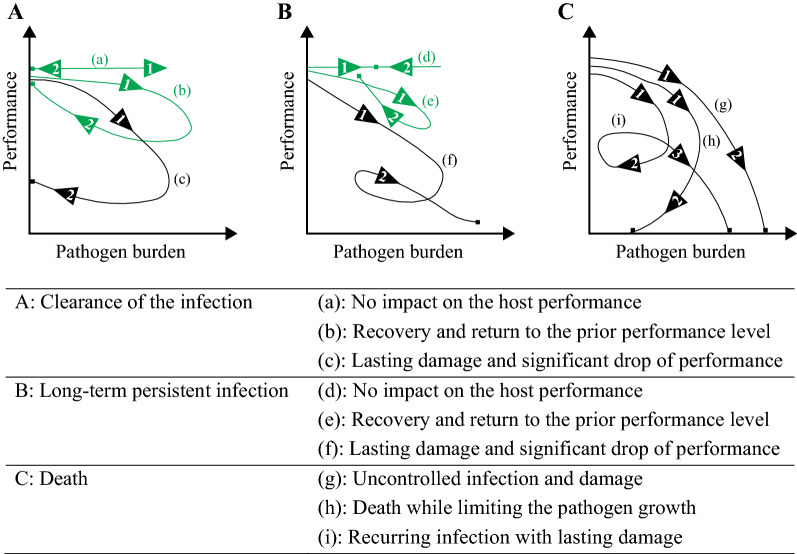
Nine pathogen burden–performance trajectory archetypes caused by disease resistance-tolerance interactions (derived from Doeschl-Wilson et al. 2012a). The trajectories can be first classified into the eventual clearance of the infection (**A**), long-term persistent infection (**B**), and death (**C**) outcomes in terms of the increased infection severity associated with the decreased disease resistance levels. Within each of these three categories, the trajectories can be further classified according to the long-term impact of infection on host performance correspond to different disease tolerance levels. Thus, for **A** and **B** categories, the host may experience little or no impact on performance (a, b, d, e) or suffer a reduction in performance (c, f). However, for the **C** category, the death of the host in response to infection can be caused by uncontrolled pathogen replication and damage (g), cumulative damage while limiting the pathogen growth (h), and cumulative damage with recurrent episodes of disease outbreaks (i). The arrows indicate the direction of trajectories over time. The squares indicate the end-point of animals, either slaughtered for the product or death

Therefore, “pathogen burden–performance trajectory” was proposed to capture the dynamic aspects of the disease process and the interaction between disease resistance and tolerance in response to infection revealed by repeated measurements of within-host pathogen burden and performance over time (Doeschl-Wilson et al. 2012a; Schneider 2011). Adapted to the context of disease resistance and tolerance interactions, Doeschl-Wilson et al. (2012a, b) described nine major classes of trajectory categories (Fig. 1). These nine categories are expected to cover all situations of livestock diseases as most pathogen burden–performance trajectories can be merged and represented by one of the archetypical curves, although the actual shapes of individual trajectories within each category may differ considerably from each other (Doeschl-Wilson et al. 2012a; Schneider 2011).

Four trajectory categories (a, b, d, e) highlighted in green in Fig. 1 are of particular interest for profitable production because the overall performance of the host is maintained at an undepressed level when it is infected. Such an attribute is defined as disease resilience and may be caused by an animal’s ability to maintain performance regardless of the change of pathogen burden (a and d in Fig. 1) or the ability to cope with the perturbation and return to pre-challenge status (b, e in Fig. 1) (Albers et al. 1987; Colditz and Hine 2016; Friggens et al. 2017). Thus, one may aim to achieve an optimal trajectory in the pathogen burden–performance space to improve the productivity of the pig industry in the face of diseases, which would correspond to breeding for disease resilience, a combined optimal balance of tolerance and resistance mechanisms (Anacleto et al. 2019; Doeschl-Wilson et al. 2012a). However, exploring a practical way to quantify individual trajectories and summarize the information into phenotypes is a significant hurdle that may hinder the subsequent genetic analyses for breeding disease resilience in the pig industry.

Current studies of disease resilience have focused on one-dimensional resilience trajectories regardless of pathogen burden because resilient animals can maintain performance despite the presence of different disease agents. Multiple traits derived from production and fitness performance data have been explored for the operational measurement of disease resilience in livestock production in challenge tests. Firstly, the deviation of production traits has been explored to measure disease resilience. In this case, more resilient animals are expected to show minor deviations in performance compared with susceptible animals because they are less influenced by infection or have the ability to rapidly recover from disease (Berghof et al. 2019b). For example, the deviations of wool growth and wool fibre diameter in Merino sheep when measured during uninfected, infected, and recovered states in relation to the parasite *Haemonchus contortus* were used as proxy traits of disease resilience to measure the depression and recovery of productivity due to infection (Albers et al. 1987). However, the heritabilities of these traits were too low (not significantly different from zero) to make tangible genetic improvement (Albers et al. 1987). In dairy cows, disease resilience measured by the fluctuation of milk yield of an individual cow per lactation was moderately heritable (0.10 to 0.24) and genetically correlated (− 0.22 to − 0.66) to udder health, incidence of ketosis, and overall cow longevity (Elgersma et al. 2018; Poppe et al. 2020). In layer chickens, parameters that indicate the deviation of body weights over time from an individual were investigated as resilience phenotypes with heritability estimates ranging between 0.09 and 0.11 (Berghof et al. 2019a). Variation of feed intake and feed duration were also tested as proxy traits of disease resilience of growing animals during infection because a more significant reduction in intake is often observed in susceptible animals during clinical disease (Sandberg et al. 2006).

Immune traits have also been proposed as proxy traits of disease resilience because immunocompetence (the ability to produce effective and appropriate immune responses) is closely associated with disease severity and tissue damage (Hine et al. 2014; Wilkie and Mallard 1999). Thus, immunocompetence may be a key player in balancing between immune responses and tissue damage to maintain an animal’s performance and productivity in response to the disease challenge. Multiple immune traits, such as antibody titers, immune cell counts, and cytokine levels, have been explored and demonstrated as candidate measures of disease resilience in pigs based on the relatively high level of estimates of their heritability and genetic correlations with production traits (Clapperton et al. 2008, 2009; Flori et al. 2011; Wilkie and Mallard 1999). For example, the concentrations for peripheral blood mononuclear leukocytes of pigs raised on low health status farms for 60 days were heritable (0.18 to 0.71), and a significant genetic correlation (− 0.46) was found between monocyte concentration and average daily gain (Clapperton et al. 2008, 2009).

In addition, mortality as a fitness trait has also been explored for breeding disease resilience (Knap 2005). However, mortality is a notoriously difficult trait to use for breeding as it typically has a low incidence and heritability (Knol et al. 2016). Typically, average mortality in a wean-to-finish pig barn is expected to be 6% to 8%, and the finishing barn mortality may only average 4% to 6% (Stalder 2013). Mortality can also have many causes other than infection. Therefore, precise tracking of mortality is required for it to be useful in addressing disease resilience. For example, date and reason for death needs to be recorded along with the pig ID or the tag number, and sometimes necropsy may be needed if cause is unclear. For these reasons mortality recording is very costly and laborious if it is to be useful for the purpose of selection.

As indicated earlier there are several different options for proxy traits of pig disease resilience in pigs. However, another hurdle that hinders selection for disease resilience is that many of these proxy traits need to be determined when disease is present as they are not expressed in the high-health environments where the selection of elite breeding animals occurs (nucleus herds). One potential approach is to use of vaccination or the application of mitogens (e.g. lipopolysaccharide and phytohemagglutinin) on healthy animals in nucleus herds to stimulate the immune system and thereby assess immune responses involved in disease resilience. The immune response test developed by Mallard and colleagues and used in dairy cattle is one possibility that is being investigated for application in pigs (Schmied et al. 2018). Indicator traits of disease resilience that can be collected on young selection candidates in nucleus farms where most phenotype recording and selection take place would be the most cost-effective approach.

An alternative is to use genomic selection to select for disease resilience based on data recorded on relatives of nucleus selection candidates grown in commercial conditions. Genomic prediction allows for the early selection of elite breeding animals from the high health nucleus herds without any records under disease. This is because it predicts the genomic estimated breeding value of an animal by summing up all SNP marker effects over the whole genome (Meuwissen et al. 2001; Samorè and Fontanesi 2016). The marker effects can be estimated as a regression of phenotypes on genotypes of relatives of nucleus selection candidates grown in commercial conditions in the face of disease challenge.

Overall, disease resilience results from combined optimal balance of tolerance and resistance mechanisms. Selection on disease resilience has been recognized as a pragmatic way of increasing disease resistance and tolerance to infection in the absence of records of pathogen burden (Mulder and Rashidi 2017). Making genetic improvement of disease resilience may be the most useful way forward for the pig industry to cope with the complex disease challenge caused by multiple different pathogens.

### A natural disease challenge model (NDCM) for improving disease resilience

Controlled challenge studies with specific pathogens had shown the potential to select for reduced susceptibility to pathogens such as PRRSV (Boddicker et al. 2014a; Serão et al. 2016; Waide et al. 2018). However, it was not known what would happen in commercial production when animals are faced with multiple disease agents. As a result, a consortium of international breeding companies (PigGen Canada) and researchers primarily in Canada and the US decided to address some of the challenges described above, especially in terms of the number of diseases challenging pig producers around the world. A wean-to-finish natural disease challenge model was established at a test station at Deschambault in the province of Québec, Canada, to mimic commercial production environments (Bai et al. 2020; Putz et al. 2019). Briefly, batches of test pigs were sourced from healthy multiplier farms from multiple genetic suppliers in rotation. Every 3 weeks, a batch of 60 or 75 high health weaned test pigs were introduced into the facility in a continuous flow with approximately 3300 pigs entering between 2015 and 2019.

The first phase consisted of a quarantine unit to mimic the high-health status at genetic nucleus farms. Pigs were then moved to a challenge unit, established by introducing pigs from commercial units known to have different pathogens present with the first few batches of healthy pigs. In this way each subsequent batch of pigs was challenged by exposure to older pigs introduced into the challenge nursery 3 weeks earlier. After 1 week together the older pigs were moved to a 16-week grow-to-finish stage.

Common disease-causing pathogens found in commercial farms were the primary target of the NDCM, including multiple strains of PRRSV and swine influenza A virus, various respiratory and enteric bacterial pathogens (such as *Mycoplasma hyopneumoniae*, *Haemophilus parasuis*, *Brachyspira hampsonii*, *Salmonella enterica* serovar *typhimurium*, and *Streptococcus suis*), and parasites including *Cystoisospora suis* and *Ascaris suum*. In addition, other pathogens including PCV2 (controlled by vaccination), porcine rotavirus A, *Erysipelothrix rhusiopathiae*, *Staphylococcus hyicus*, and some undefined minor pathogens were also present. Not all pigs were exposed to the same types or doses of pathogens as the disease pressure can vary on a batch or season basis, except every batch was confirmed to have been exposed to PRRSV. The NDCM was operated with careful veterinary oversight and group and individual antibiotic treatments were given to the animal as necessary to keep morbidity and mortality within agreed levels to ensure appropriate animal care and adherence to humane end points. Thus, the NDCM is a combination of the circulating pathogens, together with the environment, management, and veterinary strategies present, as would be the case on a commercial farm.

Importantly, the NDCM allowed the collection of different measures of resilience including growth and treatment rates, and feed and water intake. In addition, metabolites in plasma and a range of health and immune traits, including health condition scores (HScore), complete blood count (CBC) and natural antibody level (NAb) were also collected from the NDCM for multiple genetic studies of disease resilience (Bai et al. 2020; Chen et al. 2020; Cheng et al. 2020; Dervishi et al. 2021). Most traits were found to be moderately heritable except for HScore which was not significantly different from zero (Putz et al. 2019; Bai et al. 2020; Chen et al. 2020; Cheng et al. 2020; Dervishi et al. 2021). The potential utility of these traits was assessed according to their genetic correlations with several economically important production traits and measures of resilience, such as average daily gain, grow-to-finish growth rate, average daily feed intake, feed conversion ratio, residual feed intake, and carcass traits.

Some of the phenotypes show promise as indicator traits for improving reisilience. For example, higher plasma concentration of oxoglutarate but lower concentration of creatinine in healthy pigs were found to be genetically correlated with lower treatment incidence and mortality in response to disease challenge, respectively, which may lead to improved disease resilience (Dervishi et al. 2021). Higher NAb titers for immunoglobin G binding peptidoglycan (PDG-G) in the quarantine stage was found to be associated with the higher individual treatment incidence, indicating that higher PDG-G may lead to lower disease resilience (Chen et al. 2020). In addition, multiple phenotypes collected in the challenge unit have also been explored as candidate proxy traits of disease resilience that can be collected from outbreaks in commercial herds to further develop them as measures for genomic selection of disease resilience. For example, lower feed intake and feed intake duration variability might associate with higher disease resilience based on lower mortality and treatment incidence, and higher average daily gain and carcass weight (Cheng et al. 2020; Putz et al. 2019). A few CBC traits, such as increased lymphocyte concentration at 2-weeks post-infection but decreased levels of neutrophil concentration and red blood cell distribution width (variability in the size of red blood cells) at 6-weeks post-infection, were found to be associated with higher disease resilience in terms of higher growth rate and lower treatment incidence (Bai et al. 2020).

Further validation and evaluation of cost efficiency are required for these indicator and proxy traits of disease resilience before they can be applied. Continuous monitoring and collecting data from nucleus and commercial farms will be necessary to re-estimate genetic parameters of these traits and their associations with disease resilience. Pig signals (e.g. behavior, sound, smell, etc.) and clinical signs, and recording of morbidity and mortality need to be continuously monitored. Production and fitness traits and animal behaviour in commercial conditions also need to be closely monitored and regularly recorded. All these together will help ensure the animals are being selected and bred in the right direction and avoid potential antagonistic relationships or undesirable trade-offs between the improvement of disease resilience and the animal’s productivity and welfare.

### Multi-omics data for improving disease resilience

Disease resilience is a complex trait composed of multiple biological functions, although it has been studied using genomic data, the underlying biological mechanisms are still a black box, impacting the significance and accuracy of the selection of disease resilience. Therefore, information on different levels of post-genomic regulation also needs to be explored. The remarkable development of high-throughput omics technologies provides an opportunity to dissect complex traits into biologically better defined components (Kasper et al. 2020; Suravajhala et al. 2016). Information from different omics techniques, such as gene polymorphisms (genomics) and quantification of gene transcripts (transcriptomics), proteins (proteomics) and metabolites (metabolomics), are integrated to obtain a more complete picture of the processes that result in the observed phenomenon (Kasper et al. 2020; Suravajhala et al. 2016). Furthermore, microbiome data should also be included for improving disease resilience as the host microbiome also plays a significant role in enhancing host functions and contributing to host health and fitness (Mueller and Sachs 2015). For example, the early-life gut microbiota profile of pigs could potentially predict vaccine response against *Mycoplasma hyopneumoniae* (Munyaka et al. 2019, 2020), an important pathogen involved in porcine respiratory disease complex together with PRRSV. Host genetics has now been recognized to play an important role in influencing the gut microbiota in both humans and livestock (David et al. 2019; Khachatryan et al. 2008; Li et al. 2019). This could open up the potential for future research to characterize the composition and function of a “healthy” pig gut microbiota and modulation of the microbiota through selective breeding together with nutrient and management options for improving health.

The understanding of the mechanisms of tail-biting behaviour in group-housed pigs has been significantly improved through studies using multiple omics (genomics, transcriptomics, and metabolomics) techniques (Brunberg et al. 2013; Palander et al. 2013; Ursinus et al. 2014; Valros et al. 2015; Wilson et al. 2012). Accordingly, analyses of multi-omic data, including genomic, transcriptomic, proteomic, metabolomic, and microbiome data, collected from the NDCM are continuing to explore heritable and easily measurable traits and biomarkers for selecting disease resilience. Meanwhile, it will also improve our understanding of biological mechanisms associated with disease resilience and how causative genetic polymorphisms give rise to different phenotypes. Furthermore, integrating information of these multi-omics data and environmental conditions is on the way to explore a robust prediction of disease resilience through the application of machine learning.

### What is the next step

The breeding of livestock species has shifted from primary production-only goal traits to balanced breeding goal traits that aim to simultaneously improve production, efficiency, and health traits (Berghof et al. 2019b). Genetic improvement of resilience fits within the philosophy of balanced breeding because it may improve an animal’s ability to cope with disease challenges while maintaining a relatively undepressed production and fitness performance. Consequently, it can reduce production losses, costs of health treatments and uses of antibiotics, veterinaran costs, and labour costs of caring for sick animals. The economic value of improving disease resilience in pigs could be high with an increasing number of animals per farm (Berghof et al. 2019b; Knap and Doeschl-Wilson 2020). However, there is still more work to do before the selection of disease resilience can be implemented into the pig breeding programs.

### Disease resilience and its effect on the infection itself

To date, experimental studies of disease resilience in response to a polymicrobial challenge were primarily defined and quantified by animal productivity and performance regardless of pathogen burden. This property of disease resilience makes it a potentially practical strategy for the pig industry as it focuses on productivity and performance resulting from the interaction of disease resistance and tolerance rather than pathogen burden. This is because recording pathogen burden can be significantly more difficult and expensive compared with collecting animal performance traits. As discussed here pigs can be challenged by multiple pathogens in commercial farms, and the pathogens can be distributed non-uniformly throughout multiple different cells, tissue, or organ compartments of the body, many of which are challenging to sample (Cunnington 2015). Typically, measuring pathogen burden is constrained to the use of samples that are readily accessible such as blood, urine, and feces, by assuming them to represent total pathogen load (Cunnington 2015). In addition, multiple different samples and methods may be needed when the animals face polymicrobial challenge, for example, reverse-transcription or real-time polymerase chain reaction and enzyme-linked immunosorbent assay for some viruses and bacteria, plating and culture for bacteria, or fecal egg/worm count for parasites. Although these methods are well established, skilled operators and specialized equipment are often required with the associated additional cost.

Resilient animals can harbour high pathogen load shown as trajectory categories d and e in Fig. 1 when the selection was made based on performance in the absence of pathogen burden. Typically, the effect of selection for disease resilience on the infection itself, including disease transmission and pathogen evolution, remains unknown.

As indicated above although selective breeding for disease resistant animals that exert control on pathogen burden can limit disease transmission in the population, it is not necessarily practical because of the difficulty of monitoring pathogen burden. For example, there is no concern on disease transmission when the basic reproduction ratio (R_0_, expected number of cases directly generated by one case in a completely susceptible population) of infection is lower than 1 (Bishop and MacKenzie 2003; Heffernan et al. 2005). In this case, less than one naïve pig could be infected during the infectious period of a pig on average, and the disease will die out on its own (Heffernan et al. 2005). An epidemic can arise in the population when R_0_ is higher than one, thus, more than one naïve pig gets infected during the infectious period of a pig, and disease will propagate to susceptible animals (Bishop and MacKenzie 2003; Heffernan et al. 2005). However, the resulting epidemic may not necessarily be a concern for the profitability of the pig industry as long as the animals have been selected for disease resilience and can maintain high productivity regardless of pathogen burden.

Nevertheless, pathogen burden and disease transmission should not be overlooked in the pig industry, especially for zoonotic pathogens. Pigs and pork products can have high zoonotic pathogen burden (e.g. *Salmonella* spp*.*, *Escherichia coli*, and many parasites) when disease resilient animals are selected based on performance without measuring pathogen burden, shown as trajectory categories d and e in Fig. 1. Such high zoonotic pathogen burden can threaten human health, especially for farmers working in the pig industry and consumers (Djurković‐Djaković et al. 2013; Hill et al. 2010; Honish et al. 2017; Prendergast et al. 2009; Tseng et al. 2014). In this case, breeding for resilient animals that demonstrate clearance of the infection (trajectory categories a and b in Fig. 1) is of particular importance for disease eradication. Thus, in addition to longitudinal production and fitness performance, individual time-series data of pathogen burden may need to be recorded for two-dimensional resilience trajectories (Knap and Doeschl-Wilson 2020). Both Knap and Doeschl-Wilson (2020) and Mulder and Rashidi (2017) indicated the importance and advantage of including the pathogen burden in breeding for disease resilience. However, novel analytical approaches to derive reliable descriptors that capture the trajectory characteristics and can be lent to routine genetic evaluation are required for implementing the dynamic trajectories in practical breeding programs (Doeschl-Wilson et al. 2012a; Knap and Doeschl-Wilson 2020). To date, the dynamic trajectory has been mainly described and used with well-controlled challenge tests targeting a specific pathogen, and its value to practical breeding in the pig industry is yet to be determined (Lough et al. 2015; Rath et al. 2018; Torres et al. 2016).

Furthermore, pathogen evolution of escape mutations in response to selection on the host is often raised as a risk with genetic disease control strategies. Although the hosts evolve simultaneously through the selection, pathogens could adapt to the environment more rapidly than the hosts due to their much shorter generation intervals and horizontal gene transfer in bacteria (Carrillo-Bustamante et al. 2015). Pathogen evolution with the selective breeding of disease resistance has been commonly discussed as the immune mechanisms employed by disease resistant animals to defend against pathogens can cause harmful effects on pathogen fitness and impose selection pressure on pathogens (Margolis and Levin 2008). In contrast, disease tolerance may form stable host–pathogen associations (mutualism) that give neither host immune mechanisms nor pathogen virulence an evolutionary incentive because there are no competitive mechanisms (Little et al. 2010; Roy and Kirchner 2000).

The effect of selection for disease resilience on pathogen evolution remains largely unknown. However, with the contribution of both disease resistance and tolerance, disease resilience is expected to balance the benefits and concerns to some degree, showing a tendency to fuel the “arms race” between host and pathogen but less likely to be as significant as disease resistance. Therefore, this aspect will need to be further evaluated in terms of whether resilience can lead to more virulent and hard to control pathogens.

Overall, continuous and routine monitoring of individual pathogen burden and its associated morbidity and mortality need to be considered, which will help ensure the animals are being selected and bred in a sustainable way regarding pathogen transmission and evolution. Cost-efficient technologies are required to reduce the cost and labour for routine measures of pathogen burden in commercial farms where the disease challenge can be caused by multiple different pathogens. Therefore, in addition to the endemic pathogens that have been studied in the NDCM, surveillance and continuous research of disease resilience to other emerging pathogens (e.g. ASF and PED viruses to the Canadian pig industry) is also important in the future.

### Breeding general resilience: robustness

During life on farm, throughout transport, and at slaughter, pigs can be exposed to many potential challenges and stressors. Besides disease challenge, pigs face multiple non-infectious environmental challenges, such as stress associated with social status or hierarchy, extreme climatic conditions (heat stress), poor air quality, and low feed quality (Colditz and Hine 2016; Knap 2005). These factors show adverse effects on the physiological, behavioural, and affective states of animals, resulting in reduced production, poor health and bad welfare. For example, the poor air quality in a farm can lead to a significant reduction in the growth performance of grower pigs. It has been seen that the average daily gain of grower pigs can be reduced by 12% to 30% when aerial ammonia levels in the barn increased from 50 to 150 ppm (Drummond et al. 1980). Usually, a pig farm can face multiple challenges simultaneously that further intensify adverse effects on animal performance. For instance, high ammonia exposure together with infection by *Ascaris suum* (one of the most common nematode parasites of pigs) was found to result in a significantly higher percentage reduction (61%) in the average daily gain of pigs compared to either ammonia-exposed (32%) or infection (28%) alone (Drummond et al. 1981). Once the animals are under stress, the stress hormones (glucocorticoids) could disrupt the homeostasis of immune cells by inducing suppression or enhancement of innate immune response and cytokine production (e.g. interleukins-4, -5, -6, -12, and interferon-γ), and therefore, disturb immune function and increase susceptibility to disease (Salak-Johnson and McGlone 2007; Sapolsky et al. 2000). Moreover, since climate change will impact the pig production sector in addition to its spread to hotter regions, heat stress will become a very significant concern associated with decreased productivity and increased mortality (Cross et al. 2020; Gabler and Pearce 2015; Knap 2005; Mayorga et al. 2018).

In line with the above trends, the breeding goal will need to be further extended in the longer term from disease resilience to a more general resilience in order to maintain the sustainability of the pig industry. Therefore, robustness is used to describe high resilience to perturbations in an intensive pig production environment, including both infectious and non-infectious challenges caused by pathogens, weaning, housing conditions, social environment, heat, and etc. (Knap and Bishop 2000; Knap 2005). Like disease resilience, robustness is also a complex trait that is hard to target for breeding. Robustness includes multiple biological functions, such as sensing, processing and regulation of responses to and adaption to environmental stimuli and changes (Colditz and Hine 2016). The sensors and receptors involved in robustness are found in the peripheral nervous system, and throughout the body and immune system, respectively (Colditz and Hine 2016). Again, easy and inexpensive descriptors of robustness are required for practical breeding for this goal. Multiple variables, such as body temperature changes, heart rate variability, normality of circadian ethogram, feed intake variability, growth and principle production variables, immune responsiveness, normality of demeanour and vocalization, are likely to help define and measure robustness (Colditz and Hine 2016). With increasing advocacy for precision pig farming and burgeoning research of monitoring technologies, biosensors, and other high-throughput phenotyping technologies, more time-series data and performance traits will become routinely and readily available to assess robustness and also improve the management and control of environmental challenges (Benjamin and Yik 2019; Koltes et al. 2019; Neethirajan 2017). The continuous development of new technologies for automated data recording in the pig industry will enhance the pig specialist’s eyes, ears, and nose to capture “pig signals” and help to manage a larger population with fewer hands in everyday farming.

## Conclusions

Including disease resilience in breeding programs has great potential to maintain productivity and reduce the antimicrobial use of the pig industry in the face of diseases challenge, in addition to the development of conventional methods of biosecurity and vaccination. However, breeding disease resilience is not a straightforward process due to the difference between selection and production environments. Moreover, disease resilience itself is a complex trait that is hard to measure for selection. Studies are ongoing to explore easy and inexpensive indicators, proxy traits, and biomarkers, and their use for genomic selection, for the practical breeding of disease resilience. The genetic correlations between disease resilience traits with production and fitness performance also need to be evaluated to make the breeding goal more sustainable by including different types of traits and selecting all into the desirable direction. With the remarkable development of high-throughput omics and phenotyping technologies and mathematical and statistical methods adapted to these data, together with genome editing, there are exciting opportunities for the pig industry to overcome the above obstacles and breed disease resilience and robustness to current and future hard-to-manage diseases and environmental changes. Consequently, it will improve the productivity and sustainability of the pig industry and support the development of the industry towards high concentration and globalization required to supply demand for highly nutritious animal protein.

## Data Availability

Not applicable.

## Abbreviations

ASF: African swine fever
CBC: Complete blood count
CD163: Cluster of differentiation 163
*E. coli*: *Escherichia coli*
FUT1: Alpha-(1,2) fucosyltransferase
GBP5: Encoding guanylate-binding protein 5
HScore: Health condition scores
NAb: Natural antibody level
NDCM: Natural disease challenge model
PDG-G: Immunoglobin G binding peptidoglycan
PCV2: Porcine circovirus type 2
PED: Porcine epidemic diarrhea
PRRSV: Porcine reproductive and respiratory syndrome virus
SNP: Single-nucleotide polymorphism
SRCR5: Scavenger receptor cysteine-rich domain 5
TGEV: Transmissible gastroenteritis virus
